# Warmth and reciprocity with mothers, and young children's resilience to exposure to community violence in Colombia: findings from the La Sabana Parent–Child Study

**DOI:** 10.1111/jcpp.13629

**Published:** 2022-05-09

**Authors:** Diana Obando, Nicola Wright, Jonathan Hill

**Affiliations:** ^1^ 27989 Department of Psychology Universidad de La Sabana Chia Colombia; ^2^ 5289 Department of Psychology Manchester Metropolitan University Manchester UK; ^3^ 6816 School of Psychology and Clinical Language Sciences University of Reading Reading UK

**Keywords:** Resilience, callous‐unemotional traits, exposure to community violence, positive parenting, negative parenting, low‐ and middle‐income country

## Abstract

**Background:**

Exposure to community violence is common worldwide and is associated with emotional and behavioural problems in children. Little is known about sources of resilience. Building on our previous work on the contribution of callous‐unemotional (CU) traits to child aggression in Colombia, we examined whether positive parenting is protective for children whose families are exposed to community violence.

**Methods:**

Families were recruited from three demographically contrasting regions of Colombia. The sample comprised 235 children aged 3.5 years and their mothers, of whom 220 (93%) were followed up at age 5.0 years. Positive parenting was assessed as the average of maternal warmth and reciprocity, and as praise, and negative parenting as the average of negative affect and conflict seen in video recordings of standardized procedures. CU traits and oppositional defiant disorder were assessed by maternal report at ages 3.5 and 5.0 years, and mothers reported exposure to community violence over the 18 months between assessments. A range of potential confounds was included in adjusted analyses.

**Results:**

In the families who were exposed to community violence, but not in the unexposed, maternal warmth and reciprocity were associated prospectively with lower CU traits (interaction, *p* = .007). In the exposed group maternal warmth and reciprocity explained 10% of the variance (β = −.34, *p* = .001). Maternal praise was not associated with CU traits. Maternal negative parenting predicted higher CU traits as the main effect but not in interaction with community violence exposure.

**Conclusions:**

Maternal warmth and reciprocity with young children may promote resilience in the face of community violence. Programmes to enhance these protective processes may be needed especially where prospects for reducing community violence are limited. The centrality of parents for these children highlights the plight of those exposed to community violence, and also either separated from parents or orphaned.

## Introduction

Low concern for others’ distress and lack of guilt regarding the impact of one’s harmful behaviour, commonly referred to as ‘Callous‐unemotional’ (CU) traits, are associated with a distinct vulnerability for child conduct problems (Frick, Ray, Thornton, & Kahn, 2014). These traits are an important focus of research because they commonly persist and give rise to a wide range of adverse outcomes later in life (Hill & Maughan, 2015). Most of the evidence regarding processes contributing to the development of CU traits comes from countries characterised by the World Bank as ‘High‐Income’ Countries (HICs) and little is known about the shared and distinct risks for CU traits in ‘Low and Middle‐Income’ countries (LMICs). We examine risk from exposure to community violence, a risk external to the family that is experienced worldwide but is particularly common in Colombia. Based on existing HIC evidence demonstrating a promotive and protective role of positive parenting, we examine whether positive parenting at age 3.5 years is protective for CU traits at age 5.0 years in children whose families are exposed to community violence.

### Role of parenting in CU traits

Developmental hypotheses and studies of parenting early in life and CU traits point to the role of positive parenting. We focus on two dimensions: (a) maternal warmth and reciprocity, and (b) positive reinforcement of prosocial behaviour. Warmth and sensitivity (in early childhood) or reciprocity (in later childhood) are thought to reduce CU traits by helping the child to develop emotional attachments to others and to learn to recognize and respond to signs of distress in others (Dadds et al., 2014; Wright, Hill, Sharp, & Pickles, 2018). Originally, maternal sensitivity was conceptualised as responsiveness to infant cues (Ainsworth, Blehar, Waters, & Wall, 1978), but in the majority of studies, responsiveness has been combined with warmth to create maternal sensitivity composites (Mesman & Emmen, 2013). We examined warmth and sensitivity in a study from infancy and showed each made independent contributions to lower CU traits assessed from age 2.5 to 5 years (Wright et al., 2018). In other studies from infancy or toddler age using maternal sensitivity and warmth composites, prospective associations with CU traits assessed up to 6.0 years have been shown (Bedford et al., 2017; Wagner, Mills‐Koonce, Willoughby, Zvara, & Cox, 2015). Of these studies, only Wagner et al examined the role of parenting dimensions uniquely for CU traits by controlling for associated conduct problems.

Positive reinforcement of prosocial behaviours may also be relevant to reducing CU traits by capitalising on the child’s reward dominant response style to help promote prosocial behaviour. In a sample of 561 adopted children, observed positive reinforcement at age 18 months was associated with lower CU traits after accounting for conduct problems at age 2 years (Waller et al., 2017). However, and in contrast, in another large prospective study (*n* = 731) using observational and self‐report measures of positive reinforcement and harsh parenting at age 2 years, harsh parenting but not positive reinforcement predicted CU traits at ages 3 and 4 years (Waller et al., 2012). This study did not examine the unique contribution of parenting to CU traits, by controlling for associated conduct problems. Importantly, positive reinforcement in infancy has been shown to be protective for CU traits, independent of conduct problems, in the context of biological risks for CU traits (Hyde et al., 2016; Waller et al., 2017). In this study, we examine whether positive parenting, assessed as the average of warmth and reciprocity, and as positive reinforcement, confers resilience to risk from outside of the family, community violence. By controlling for associated oppositional defiant disorder (ODD) behaviours we test whether this is unique to CU traits.

### Measurement of CU traits

A small number of studies have provided evidence for the reliability and validity of established measures of CU traits with older children and adolescents in LMICs (Amador & Padrós, 2019; Wang et al., 2019). In a previous publication from *La Sabana Parent–Child Study,* we have reported that the factor structure of the Inventory of Callous Unemotional Traits (ICU; Frick, 2004), is similar to that found in preschoolers in HICs (Kimonis et al., 2008) and provided evidence for incremental validity in relation to aggression, supporting the validity of the CU traits construct and measure in an LMIC setting. Furthermore, in a test of cross‐cultural robustness, we showed that the prediction from CU traits to aggression at 5.0 years was significantly greater in the presence of high aggression at age 3.5 years in the Colombian sample and also in the UK Wirral Child Health and Development Study (Obando et al., 2021).

### Exposure to community violence

Although the evidence summarised so far points to individual and intrafamilial contributions to CU traits, in many children, antisocial behaviours develop in the context of wider contextual factors, particularly community violence. The Report on Violence and Health from the World Health Organisation (WHO, 2002) describes community violence as a public health concern, with adverse effects on children’s emotional, social, academic, behavioural, and cognitive processes (Sharkey, Schwartz, Ellen, & Lacoe, 2014). Associations between community violence in the form of muggings, knifing, shooting, and child adjustment problems, both internalising and externalising, have been extensively documented and well‐replicated across a large number of studies (e.g. Fleckman, Drury, Taylor, & Theall, 2016; Mohammad, Shapiro, Wainwright, & Carter, 2015).

The evidence on the link between community violence and CU traits is much more limited. However, understanding the role of this important environmental risk for violence in the development of CU traits is a critical gap in the literature given the role of CU traits in conferring risk for future violent behaviour. Psychological and biological mechanisms that might link community violence to CU traits have been proposed, including that exposure leads to desensitization to the effects of violence on victims (Davis, Ammons, Dahl, & Kliewer, 2015), or to a coping strategy involving a reduction in processing of others’ social and emotional cues (Hill, Murray, Leidecker, & Sharp, 2008), or downregulation of physiological and neurobiological responsivity (Hill & Maughan, 2015; Saxbe et al., 2018). Two studies have examined the association between exposure to community violence and CU traits in adolescence. Exposure to community violence was associated with CU traits in a cross‐sectional study of adolescent detained boys (Kimonis et al., 2016) and a prospective study of low‐income adolescents (Davis et al., 2015).

As far as we are aware, no studies have examined the association between community violence and CU traits in children. The majority of research on broader behavioural problems has been with school‐aged children and adolescents, so relatively little is known about whether community violence similarly affects preschool children who may be more protected in their families than older children. The existing evidence is inconsistent. Linares et al. (2001) reported a cross‐sectional association between community violence and maternal report of externalising behaviours in 160 children aged 3–6 years, which was nonsignificant after controlling for maternal depressive symptoms. Shahinfar, Fox, and Leavitt (2000), also using a cross‐sectional design, showed that ‘mild’ but not ‘severe’ community violence was associated with higher parental reports of aggression in 155 preschoolers. By contrast, in a prospective study of 625 South African children, community violence assessed at age 5 years was associated with parent‐reported aggression 1 year later (Barbarin, Richter, & DeWet, 2001).

Studies of younger children also bring out issues of measurement which may give rise to inconsistencies. Unlike older children and adolescents, where self‐report is likely to be valid, this is less clear during the preschool period. Shahinfar et al. (2000) devised a method for enquiring about community violence with their preschool participants, but it showed no agreement with parent reports. Barbarin et al. (2001) asked community experts to categorize each of the communities from which children were drawn for the study using a Q‐sort procedure. In a study of young school‐age children ages 7–11 years, Cuartas and Leventhal (2020) used police records to identify exposed and unexposed children in the same areas of a city, as a way of dealing with confounding family socioeconomic factors.

### Community violence in Colombia

After almost 60 years of armed conflict, and despite the conclusion of the peace process in 2016, there are still high levels of community violence in Colombia. The Colombian population continues to be exposed to violence and displacement, particularly as a result of criminal bands and drug traffickers fighting to control Colombia’s lucrative illegal narcotics and mining industries (Ortega‐Guerrero, 2018). In 2019, almost 500,000 persons were victims of thefts, personal injuries, thefts, and homicides (Policia Nacional de Colombia, 2019), and 17% of the Colombian population in the last years has been a direct victim of the internal armed conflict (Unidad para las Víctimas, 2018).

Studies conducted in Colombia have shown associations between community violence and child mental health problems in school‐aged children. In a study conducted with 1235 children and adolescents from Bogotá, exposure to community violence was associated with both reactive and proactive aggression (Chaux, Arboleda, & Rincón, 2012). More recently, and subsequent to the conclusion of the peace process, Cuartas and Leventhal (2020) conducted a study of 404 children aged 7–11 years and found that an incident of violent crime in close proximity to children’s homes was associated with a small to medium effect size increase in children's mental health problems.

### The role of positive parenting in relation to exposure to community violence

Several studies of the association between community violence exposure and child aggression have reported moderation by positive parenting, possibly indicative of a protective effect. These include studies of resilience to exposure to military violence in Palestine (Punamäki, Qouta, Miller, & El‐Sarraj, 2011) and Israel (Slone & Shoshani, 2017), and community violence in South Africa (Barbarin et al., 2001). The protective role of positive parenting for CU traits has been examined in one study of adolescents. In the study of Davis et al. (2015), perceived parental support was associated with lower CU traits in children exposed to community violence, but it did not moderate the effect of community violence on CU traits. The protective effect of positive parenting has not been examined in a childhood sample. In this study, we predicted a protective effect of positive parenting based on the hypothesised mechanism for the effect of community violence. If exposure to community violence desensitizes children to others’ distress, then we expect warm and sensitive responses to counteract this effect by promoting the child’s capacity for empathic responding (Dadds et al., 2014; Wright et al., 2018), and positive reinforcement by promoting prosocial behaviours (Waller et al., 2017).

### Current study

In summary, prospective studies of exposure to community violence and CU traits in preschool children have not previously been conducted, either in HIC or LMIC settings, nor has the potential protective role of observed positive parenting been examined. In this paper, we examine two hypotheses. First, positive parenting at age 3.5 years, either positive warmth and reciprocity, or positive reinforcement will be associated with lower CU traits at age 5.0 years. Second, positive parenting will modify the association between community violence and CU traits consistent with a protective effect. Second, negative parenting at age 3.5 years will be associated with higher CU traits at 5.0 years. We examine (a) whether these parenting dimensions add to the risk for CU traits at age 5.0 years over and above prior risk associated with CU traits at 3.5 years, by controlling for age 3.5 years CU traits, (b) whether these associations are specific to CU traits, by controlling for ODD behaviours at age 5.0 years, and (c) whether they are evident after accounting for possible mood‐related reporter bias by controlling for maternal depressive symptoms at age 5.0 years.

## Methods

### Participants and procedure

Participants were recruited through Facebook groups with titles that suggested they were likely to include a large number of women with young children, such as ‘Latin Women League’ and ‘More Moms Colombia’. Of 304 parents who responded to the invitation, (77.3%) agreed to participate and provided full data (see Obando et al., 2021 for a full description of sampling). At recruitment mother’s mean age was 30 years (*SD* = 6.29), the children’s mean age was 3.31 (*SD* = 0.48), and 52% were boys. Follow‐up was conducted 18 months later with 220 of the families (93%) (mean age = 4.86; *SD* = 0.42, 49% boys).

Participants were from three Colombian regions, each representing different cultural and demographic features. The Central region (*n* = 96) is characterised by lower levels of poverty in the country and it is predominantly *mestizo* (mix of European and Indigenous) compared to the Pacific (*n* = 69) and Caribbean (*n* = 70) which have increased levels of poverty and high numbers of Afro‐Colombian and Indigenous inhabitants (Ministerio de Ambiente y Desarrollo Sostenible, 2013). The majority (77%) were two‐parent families, 50% of mothers and 48% of fathers had studied beyond school, and 15% of the participants lived in rural areas. Household income was operationalised based on the Colombian government system that designates 1–6 categories (1 the lowest) determined by housing conditions and basic public services (Departamento Administrativo Nacional de Estadística – DANE, 2011). As categories 1 and 2 receive subsidies from the Colombian government they were merged to represent low‐income families (45% of the study sample). Regarding participants’ ethnicity, 38% were mestizo, 9% Afro‐Colombian, 5% Indigenous, 13% from ‘other’ ethnic groups, and 35% did not identify themselves as belonging to a specific group.

### Ethical considerations

This research was approved by the Research and Ethical Committee of the Psychology Department at La Sabana University through minute number 102, 3 May 2017. Adult participants gave written informed consent.

### Measures

#### Callous‐Unemotional traits

CU traits were measured at 3.5 years and at 5.0 years by the parent‐report ICU (Frick, 2004). This inventory has 24 items each with a 4‐point scale. The Spanish version of the inventory was shared by the authors. In this Colombian sample at age 3.5 years, we replicated the best‐fitting factor structure reported by Kimonis et al. (2016) in preschool children, a 12‐item two‐correlated factor structure, and found similar internal reliability (Obando et al., 2021). For this report, we tested and replicated the same two‐correlated factor structure at age 5.0 years (Figure S1). Kimonis et al. conducted validity analyses with both the 12‐ and 24‐item totals and found similar results for both. In line with the recommendations of Ray, Frick, Thornton, Steinberg, and Cauffman (2016) we conducted the main analysis using the total 24‐item ICU scale which showed good internal consistency for this sample (age 3.5 α = .81, age 5 α = .85), but reports the results using the 12‐item total in Appendix S1.

#### Oppositional Defiant Disorder symptoms

Oppositional Defiant Disorder symptoms were assessed at the age 5.0 years using the 6‐item DSM‐IV oriented Oppositional Defiant Problems scale on the Child Behaviour Checklist age 1.5–5 years in its Spanish version (CBCL; Achenbach & Rescorla, 2001). For the present study, the scale had an internal consistency of α = .75 which is comparable to α = .69 for ODD behaviours reported previously with Colombian children (Hewitt, 2016). The CBCL factor structure has been replicated in multiple different LMICs (Resorla et al., 2012).

#### Observation of mother–child interactions

Video recordings were made of mothers and children in a standardized procedure (National Institute for Child Health and Development Early Child Care Research Network; NICHD‐ECCRN, 1999) at age 3.5 years. Parents and children were provided with three bags of toys which they are asked to play with in a preset order for over 15 minutes, and then at a preagreed signal the child was asked to tidy up the toys. Mother–child play was rated using the Parent–Child Interaction System (PARCHISY; Deater‐Deckard, 2000) generating a maternal warmth and reciprocity score (internal reliability = .73) as the average of positive affect and reciprocity, and a negativity score as the average of negative affect and conflict (Deater‐Deckard, Li, & Bell, 2016). Praise during tidy‐up was assessed using the Dyadic Parent–Child Interaction Coding System (DPICS; Eyberg, Nelson, Duke, & Boggs, 2004). Research assistants in Colombia were trained by reliable UK coders, and high agreement between them on 30 independently rated recordings in English from the United Kingdom was achieved (all ICCs >= .78). There were too few instances of labelled praise in either United Kingdom or Colombian studies for reliability analyses. In the Colombian sample, there was only one occurrence of labelled praise from a mother who also showed unlabelled praise. Unlabelled praise only was used for analysis but as this was operationalised as a binary variable due to skew it also reflects the one instance of labelled praise.

#### Exposure to community violence

Most studies of community violence have been on children and adolescents old enough to provide self‐reports. Studies of children aged 5 years and under have used a wide variety of methods, including a Q‐sort procedure to categorize levels of violence in each of the communities from which children were drawn (Barbarin et al., 2001), and a latent variable derived from several indices of local crime, social disorder, and witnessed violence (Linares et al., 2001). In view of this variability and to include exposures likely to reflect conditions in Colombia over the period from baseline to follow‐up, we devised a four‐item measure designed to reflect major violence or disruption experienced by the family. Mothers reported on whether, over the past 2 years, there had been ‘Fights’ or ‘Assassinations, kidnapping or disappearances’ in the neighbourhood, and whether the family had been directly affected by ‘Activities of guerrillas or criminal gangs’ or a ‘Victim of forced displacement’. Of the 220 followed‐up when their children were aged 5.0 years, 55 (25%) endorsed one of these items, and 26 (12%) two or more. A simple present–absent binary variable was used in all analyses.

#### Confounding variables

The Edinburgh Postnatal Depression Scale (EPDS; Cox, Holden, & Sagovsky, 1987) at age 5.0 years were included to reduce the risk of bias in reporting child behaviours by maternal mood. The EPDS has been used in previous research with Colombian females (Campo‐Arias, Ayola‐Castillo, Peinado‐Valencia, Amor‐Parra, & Cogollo, 2007) with internal reliability of α = .78. For the present sample, the internal reliability was α = .83. To deal with confounding of associations between community violence and child mental health problems by family demographic characteristics, we identified limited paternal and maternal educational levels (high school only vs. education beyond high school) and low family income (1or 2 vs. 3+ in the DANE classification) measured at age 3.5 years as potential confounds. The region, child gender, and maternal age at age 3.5 years were also included in the adjusted analyses.

### Statistical analyses

Where possible, skewed variables were transformed for parametric analyses using log transformation. Transformations of PARCHISY maternal negativity and DPICS praise did not correct skewness, while it corrected skewness of CU traits and ODD behaviours (skew statistics shown in Table S1). Bivariate analyses with these variables were conducted using nonparametric statistics, and the multivariable analyses used binary variables based on a median threshold for maternal negativity and praise. Multiple linear regression models were used to test the study hypotheses, with positive parenting, negative parenting, and community violence entered in the first block as the main effects, and two‐way interaction terms between each of the three parenting variables and community violence in the second block (unadjusted models). In the adjusted models the confounding variables were entered before the main effects and interaction terms. Models were first adjusted for age 3.5 CU traits and confounding variables, and second for age 5.0 ODD, age 3.5 CU traits, and confounding variables. Variables were centred prior to creating interaction terms. Significant interactions were explored in linear regression models examining the main effects of parenting in the exposed and unexposed groups, using the online computational tool for estimating intercepts and slopes (Preacher, Curran, & Bauer, 2006) and by plotting the associations between parenting and age 5.0 CU traits in the exposed and unexposed groups. The interactions were plotted from the adjusted regression models using the margins plot command in Stata. CU traits and parenting scores were standardised prior to plotting the interaction.

## Results

The means and standard deviations of the main variables are shown in the Table S2. Community violence was associated with low income (OR = 2.82, 95% CI 1.62–4.91, *p* < .001), limited paternal education (OR = 3.26, 95% CI 1.74–6.12, *p* < .001), and limited maternal education (OR = 1.97, 95% CI 1.08–3.61, *p* = .026). In logistic regression, both low income and limited paternal education, but not maternal education, predicted community violence, and so, to limit the number of variables in the adjusted models, we used a binary variable reflecting either one. This variable was strongly associated with exposure to community violence (OR = 3.43, 95% CI 1.93–6.10).

Bivariate associations are shown in Table S2. Notable features include that there were weak negative associations between observed warmth and reciprocity and praise and CU traits, which were significant in cross‐section but not at age 5.0 years, and a somewhat stronger and significant positive association between maternal negativity and CU traits at both ages. Reported community violence was associated with CU traits at 3.5 years but the association with CU traits at 5.0 years was small and nonsignificant.

**Figure 1:**
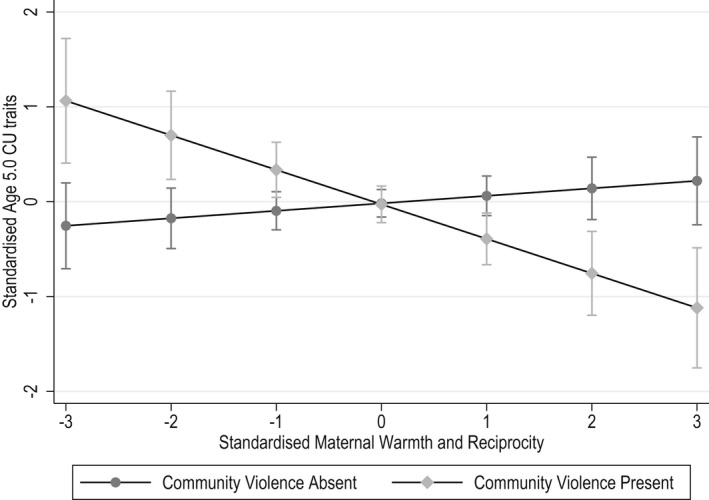
Associations between standardised maternal warmth and reciprocity and CU traits in community violence exposed and unexposed families

In multiple linear regression predicting CU traits at the age of 5.0 years, neither maternal praise nor community violence predicted CU traits, but there were main effects of maternal warmth and reciprocity (*p* = .085) and of negative parenting (*p* = .043) in the unadjusted model (shown in Table S3) with only negative parenting remaining after adjustment for confounds, age 5.0 ODD and age 3.5 CU traits (Table 1). There was a statistically significant interaction between exposure to community violence and maternal warmth and reciprocity, both prior to and after the inclusion of potential confounds, age 3.5 years CU traits, and age 5.0 ODD (Table S3; Table 1). In Figure 1, it can be seen that the highest levels of CU traits were seen in children exposed to community violence with mothers who displayed low warmth and reciprocity, while exposed children with highly positive mothers had the lowest levels of CU traits. The simple slope in the children exposed to community violence was significant and negative in direction (simple slope = −0.81(0.36), *t* = −2.28, *p* = .024) and non‐significant in the unexposed children (simple slope = 0.40 (0.25), *t* = 1.62, *p* = .106).

**Table 1:** Adjusted multiple linear regression models predicting CU traits at age 5.0 years

Variable	Overall *R* ^2^: .43			
	Δ*R* ^2^	*p*	β	*p*
Block 1	.43	<.001		
Age 3.5 CU traits			.25	<.001
Age 5.0 ODD			.39	<.001
Pacific region			−.10	.095
Caribbean region			−.09	.147
Higher maternal age			.02	.722
Low income/low education			−.08	.164
Male sex			−.04	.489
Age 5.0 maternal depression			.24	<.001
Block 2	.02	.233		
Maternal warmth and reciprocity			.01	.943
Maternal praise			.01	.927
Maternal negativity			.13	.023
Community violence			−.01	.668
Block 3	.02	.045		
Community violence × maternal warmth and reciprocity			−.20	.005
Community violence × maternal praise			.02	.843
Community violence × maternal negativity			.01	.877

In unadjusted models, among those exposed to community violence, increasing maternal warmth and reciprocity was strongly associated with decreasing CU traits (β = −.50, *p* < .001), while there was no association in the unexposed group (unadjusted models shown in Table S4). As shown in Table 2, in adjusted models, controlling for confounds, age 5.0 ODD symptoms, and age 3.5 years CU traits, the effect of maternal warmth and reciprocity in families exposed to community violence remained (−.21, *p* = .019). When using the 12‐item ICU as the outcome, the findings were very similar (Tables S5–S7).

**Table 2:** Adjusted multiple linear regression models predicting CU traits at age 5.0 in groups of families characterised by whether or not they were exposed to community violence

Variable	No community violence exposure				Community violence exposed			
	Total model *R* ^2^: .32				Total model *R* ^2^: .54			
	Δ*R* ^2^	*p*	β	*p*	Δ*R* ^2^	*p*	β	*p*
Block 1	.35	<.001			.56	<.001		
Age 3.5 CU traits			.28	<.001			.18	.061
Age 5.0 ODD			.30	<.001			.56	<.001
Pacific region			−.14	.081			−.01	.910
Caribbean region			−.11	.178			−.05	.634
Mother age			.06	.459			−.07	.389
Low income/low education			−.09	.284			−.01	.880
Male sex			−.02	.749			−.08	.315
Age 5.0 maternal depression			−.25	.004			.15	.130
Block 2	.03	.096			.05	.032		
Maternal warmth and reciprocity			.13	.097			−.22	.019
Maternal praise			.01	.950			.01	.978
Maternal negativity			.13	.089			.19	.028

## Discussion

In this prospective study of mothers and 3.5‐year old children in Colombia, as predicted, observed maternal warmth and reciprocity, at 3.5 years moderated the association between exposure to community violence over the intervening 1.5 years and CU traits at 5.0 years. Low maternal warmth and reciprocity conferred vulnerability, and high warmth and reciprocity were protective, in children whose mothers reported exposure to community violence. However, predictions of the main effects of positive parenting were not supported. Maternal negativity at 3.5 years predicted CU traits and this association was not modified by exposure to community violence. These associations were seen prior to and after controlling for age 3.5 years CU traits, indicating an incremental effect of the baseline parenting variables, and after controlling for ODD problems at age 5 years, demonstrating specificity to CU traits. Community violence exposure was not at random, with poorer families and families where fathers had limited education at higher risk. However, the observed effects were little altered in models adjusted for these demographic differences.

Our findings are similar to Waller et al. (2012) who found no significant contribution of positive parenting to young children’s CU traits after accounting for negative parenting, which itself was significantly associated with increased CU traits. The findings are also consistent with previous reports of moderation by positive parenting of risk for CU traits. However, the effect was seen for maternal warmth and reciprocity and not for positive reinforcement, in contrast to previous findings of moderation of genetic risk for CU traits by positive reinforcement (Hyde et al., 2016; Waller et al., 2016). Reidy et al. (2017) postulate that positive reinforcement is most relevant to the reduction of aggressive behaviour in the presence of CU traits, rather than in the development of CU traits themselves. In this same sample, we have shown that positive reinforcement moderates the association between CU traits and aggressive behaviour in children who are already aggressive (Obando et al., 2021).

Previous studies have not examined the role of community violence in relation to CU traits in young children, nor whether positive parenting is protective. Crucially, there is no previous evidence regarding these processes in LMICs where levels of community violence are commonly high (Westbrook & Harden, 2010). To study these processes in LMIC settings we needed to be confident that the CU traits construct and measures are valid in these settings. We have previously reported that the psychometric properties of the measure of CU traits used in this study, the ICU, are very similar in this Colombian sample to those found in HIC studies, and that the contribution of CU traits to emerging child aggression is the same across Colombian and UK samples (Obando et al., 2021). Our findings therefore cast new light on the interplay between threats from outside of the family, parental support, and the development of CU traits.

Strengths of the study included that the sample was recruited from three different regions in Colombia with contrasting demographic characteristics, via social media widely used by adults in the age range of mothers of young children, and the socioeconomic profile of the sample was similar to that of Colombia as a whole. We used a prospective design in which parenting at 3.5 years was observed, thus reducing possible shared method variance effects, and predictors of age 5.0 years CU traits could be examined after accounting for age 3.5 years CU traits. The retention of the sample at the age 5.0 assessment was high (93%).

Limitations included that participants had a higher level of education than that of the general population of Colombia. The recruitment was conducted via Facebook groups only and alternative methods of recruitment may have resulted in a more representative sample. While we accounted for some potential confounds for community violence exposure (low income and limited parental education), there may be others that we did not assess, such as violence within the home, which could have altered the findings. It is a limitation that we could not use a standard measure of community violence because available measures are self‐report questionnaires designed for use in older children. However, the strong association between the measure used here and low income and limited parental education provides support for its validity. We also collected only mother reports of community violence exposure and of CU traits, cross informant reports would remove any shared method variance issues. The lack of comparable assessments of fathers with their children is a further limitation, meaning that we were unable to determine whether paternal warmth and reciprocity are also protective, and whether it may compensate where maternal warmth and reciprocity is low (Malmberg et al., 2016).

In contrast to studies of older children, whether or not community violence is associated with elevated mental health problems in young children, remains to be established. In the studies that we reviewed, there was considerable variation (Barbarin et al., 2001; Linares et al., 2001; Shahinfar et al., 2000) and none showed prospective associations controlling for baseline problems. Two key aspects of our findings may help clarify the reasons: first, that there was not a main effect of exposure and second, that there was strong moderation by observed maternal warmth and reciprocity. The implication is that whether or not young children are affected may depend to a substantial degree, and perhaps more than for older children, on the quality of their relationships with their parents. The low levels of CU traits among exposed children who received high levels of maternal warmth and reciprocity are also noteworthy. We did not predict this effect and so we interpret with caution. However, one possibility is that children who are frightened by community violence turn to their parents for comfort more often than other children, and hence have more experiences of being cared for, thus increasing the protective effect of parental warmth and reciprocity.

Further study is required to identify which aspects of parent–child interactions may be most important for children exposed to community violence in countries like Colombia. Equally, there is a need to refine and standardise measures of young children’s exposure to community violence. Linking methods such as that used in a study from Bogotá using police records of violence in the immediate locality (Cuartas & Leventhal, 2020) to observational measures of parent–child interactions could offer a powerful way of studying children at risk and their families in Latin America and similar settings.

Our study adds to the evidence regarding the threat of community violence to children’s mental health worldwide and underlines the crucial role of the family in protecting children under these conditions. The findings indicate a need to evaluate interventions with parents in families exposed to community violence to promote positive warm interactions with their children and hence reduce the negative impact of the exposure. Furthermore, in contrast to the stable family arrangements of most participants in this study, many children are not only exposed to community violence but also separated from their parents or orphaned. They may be deprived of crucial sources of resilience.

## Supporting information


**Appendix S1**. Testing the factor structure of the age 5.0 years ICU.
**Figure S1**. Age 5.0 12‐item two‐correlated factor structure of the ICU with standardised factor loadings.
**Table S1**. Skewness and kurtosis statistics for the CU traits and ODD variables before and after transformation.
**Table S2**. Bivariate associations, Spearman’s rho between study variables and descriptive statistics.
**Table** **S3**. Summary of multiple linear regression models predicting CU traits at age 5.0 from observed maternal positivity and praise and their interaction with exposure to community violence (unadjusted model).
**Table S4**. Summary of multiple linear regression models predicting CU traits at age 5.0 from observed maternal positivity and praise in community violence exposed and no community violence exposed groups (unadjusted model).
**Table S5**. Summary of multiple linear regression models predicting the 12‐item CU scale at age 5.0 years (unadjusted for confounders).
**Table S6**. Summary of multiple linear regression model predicting the 12‐item CU scale at age 5.0 from observed maternal positivity and praise and their interaction with exposure to community violence (adjusted for confounders, age 3.5 CU traits and age 5.0 ODD). Total model R^2^ = .36.
**Table S7**. Summary of multiple linear regression model predicting the 12‐item CU traits scale at age 5.0 in community violence exposed and no community violence exposed groups, adjusted for confounders, age 3.5 CU traits and age 5.0 ODD.
