# Supplementary material for: Warmth and reciprocity with mothers, and young children's resilience to exposure to community violence in Colombia: findings from the La Sabana Parent–Child Study
Source: J Child Psychol Psychiatry. 2022 May 9;64(1):197–205. doi: 10.1111/jcpp.13629 (PMC10084088; doi:10.1111/jcpp.13629)
Supplement: Supplementary file 1 — Appendix S1. Testing the factor structure of the age 5.0 years ICU. Figure S1. Age 5.0 12‐item two‐correlated factor structure of the ICU with standardised factor loadings. Table S1. Skewness and kurtosis statistics for the CU traits and ODD variables before and after transformation. Table S2. Bivariate associations, Spearman’s rho between study variables and descriptive statistics. Table S3. Summary of multiple linear regression models predicting CU traits at age 5.0 from observed maternal positivity and praise and their interaction with exposure to community violence (unadjusted model). Table S4. Summary of multiple linear regression models predicting CU traits at age 5.0 from observed maternal positivity and praise in community violence exposed and no community violence exposed groups (unadjusted model). Table S5. Summary of multiple linear regression models predicting the 12‐item CU scale at age 5.0 years (unadjusted for confounders). Table S6. Summary of multiple linear regression model predicting the 12‐item CU scale at age 5.0 from observed maternal positivity and praise and their interaction with exposure to community violence (adjusted for confounders, age 3.5 CU traits and age 5.0 ODD). Total model R2 = .36. Table S7. Summary of multiple linear regression model predicting the 12‐item CU traits scale at age 5.0 in community violence exposed and no community violence exposed groups, adjusted for confounders, age 3.5 CU traits and age 5.0 ODD. [file JCPP-64-197-s001.docx]

Supporting Information

Appendix S1. Testing the factor structure of the age 5.0 years ICU

Replicating the 12-item two-correlated factor structure of the ICU from Hawes et al. (2014). In Obando et al. (2021) we examined the psychometric properties of the ICU in this sample and at aged 3 years and tested the competing factor structures of the ICU reported in the literature. We found that the 12 item two-correlated factor structure proposed by Hawes et al. (2014) and shown to be the best fitting factor structure for 3-year olds by Kimonis et al. (2016) fit the data best.

For this study we test whether that same factor structure shows acceptable fit to the sample at age 5 years. Following Obando et al. (2021) we used confirmatory factor analysis with weighted least squares estimation in Mplus versions 8.4 (Muthen & Muthen, 2017). The 12-item two correlated factor model fit the data well (RMSEA= .060, CFI =.97), the standardised factor loadings, which were all >.5, and correlation between the two factors are presented in Figure S1.


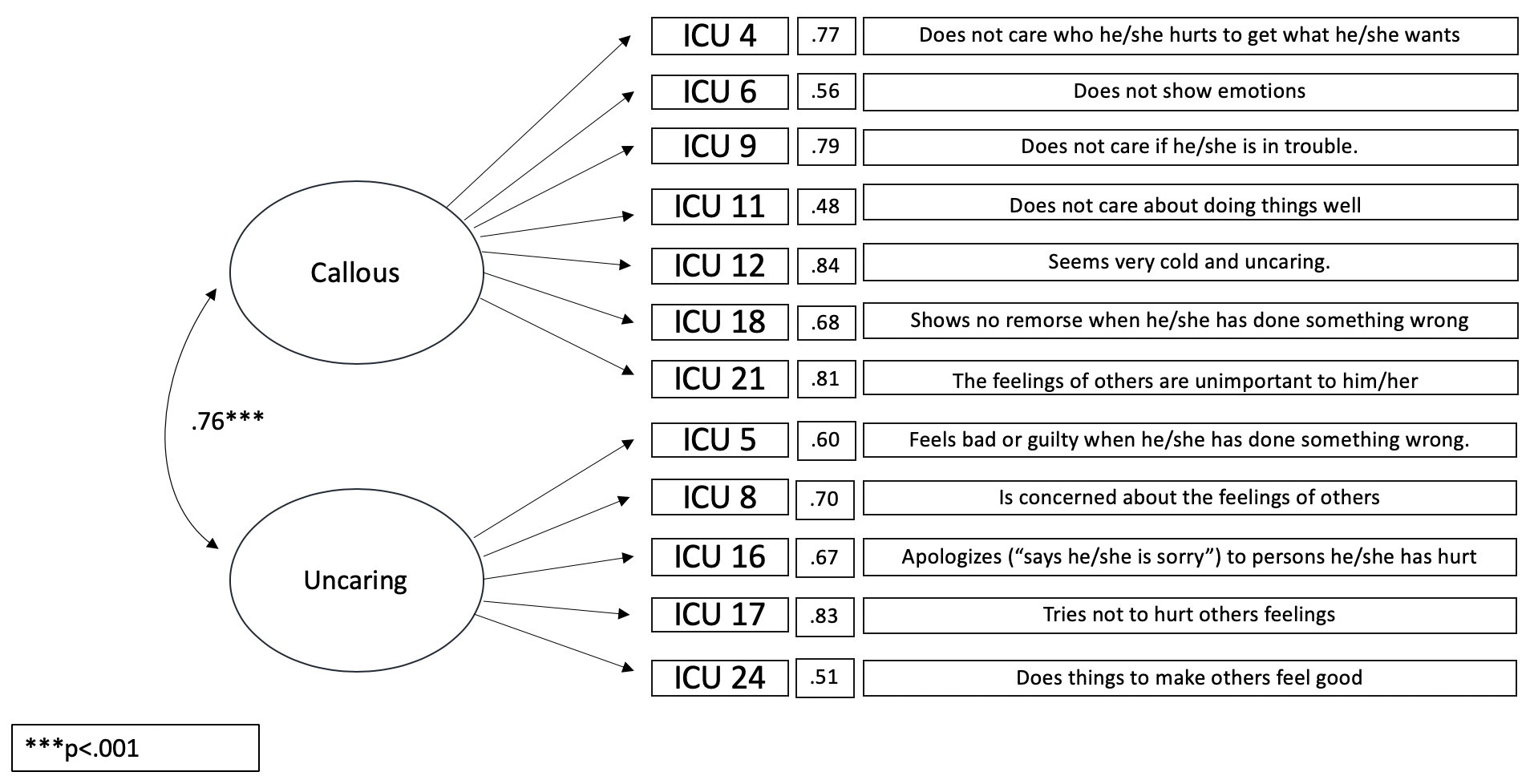


*Figure S1*. Age 5.0 12-item two-correlated factor structure of the ICU with standardised factor loadings

The internal consistency in this sample for the 12-item total is α= .71 at age 3 and α= .78 at age 5, and for the 24-item total it is α= .82 at age 3 years, and α= .85 at age 5 years.

|  | Untransformed values | | Transformed values | |
| --- | --- | --- | --- | --- |
|  | Skew (SE) | Kurtosis (SE) | Skew (SE) | Kurtosis (SE) |
| Age 3.5 CU traits | .62 (.16) | .25 (.32) | -.18 (.16) | -.05 (.32) |
| Age 5.0 CU traits | .98 (.16) | 1.52 (.33) | -.05 (.16) | .32 (.33) |
| Age 5.0 ODD | .56 (.16) | .18 (.33) | -.28 (.16) | -.65 (.33) |

Table S1: Skewness and kurtosis statistics for the CU traits and ODD variables before and after transformation

Table S2

Bivariate associations, Spearman’s rho between study variables and descriptive statistics.

|  | Positivity | Praise | Negativity | CU traits 3.5 yrs. | CU traits 5.0 yrs. | ODD 5.0 yrs | Mat. depression | Mat. age | Low-Inc/ lim. edu. |
| --- | --- | --- | --- | --- | --- | --- | --- | --- | --- |
| Praise | .12^+^ |  |  |  |  |  |  |  |  |
| Negativity | .05 | -.10 |  |  |  |  |  |  |  |
| CU traits 3.5 | -.17** | -.15* | .15* |  |  |  |  |  |  |
| CU traits 5.0 | -.11 | -.11 | .19** | .45** |  |  |  |  |  |
| ODD 5.0 | -.22** | -.04 | .06 | .39** | .54** |  |  |  |  |
| Maternal depression | .12^+^ | -.15* | .06 | .28** | .39** | .35** |  |  |  |
| Maternal age | .04 | .14* | -.03 | -.17* | -.11 | -.16* | -.33** |  |  |
| Low-income/  limited father education | .03 | -.10 | .11 | .21** | .08 | .14* | .09 | -.27** |  |
| Community violence | .03 | -.10 | .11 | .21** | .08 | .05 | .09 | -.10 | .26** |
| Male sex | .16* | .02 | .05 | .15* | .07 | .10 | -.03 | .03 |  |
| Mean | 3.27 | 1.33 | 1.36 | 16.01 | 13.85 | 3.35 | 6.17 | 30.04 |  |
| SD | 1.11 | 1.91 | .50 | 8.71 | 8.06 | 2.37 | 4.49 | 6.29 |  |
| N | 235 | 235 | 235 | 235 | 220 | 220 | 220 | 227 |  |

^+^ p <.08; * p <.05; ** p <.01; yrs.= years; Mat. = maternal; Low-Inc= Low-income; Lim. Edu.= Limited father education.

Table S3

Summary of multiple linear regression models predicting CU traits at age 5.0 from observed maternal positivity and praise and their interaction with exposure to community violence (unadjusted model)

|  |  | Unadjusted Model  R^2^: .11 | | | |
| --- | --- | --- | --- | --- | --- |
| Variable | | ΔR^2^ | p | Β | p |
| Block 1 | | .04 | .084 |  |  |
|  | Maternal positivity |  |  | -.12 | .085 |
|  | Maternal praise |  |  | -.05 | .516 |
|  | Maternal negativity |  |  | .14 | .043 |
|  | Community violence |  |  | .04 | .559 |
| Block 2 | | .10 | <.001 |  |  |
|  | Community violence X maternal positivity |  |  | -.39 | <.001 |
|  | Community violence x maternal praise |  |  | .02 | .812 |
|  | Community violence X Maternal negativity |  |  | .11 | .314 |

Table S4

Summary of multiple linear regression models predicting CU traits at age 5.0 from observed maternal positivity and praise in community violence exposed and no community violence exposed groups (unadjusted model)

|  | No community violence exposure  Total model R2: .25 | | | | Community violence exposed  Total model R^2^: .38 | | | |
| --- | --- | --- | --- | --- | --- | --- | --- | --- |
| Variable | ΔR^2^ | p | β | p | ΔR^2^ | p | β | p |
|  | .01 | .326 |  |  | .25 | <.001 |  |  |
| Maternal positivity |  |  | .11 | .198 |  |  | -.50 | <.001 |
| Maternal praise |  |  | -.06 | .498 |  |  | -.02 | .828 |
| Maternal negativity |  |  | .10 | .233 |  |  | .22 | .032 |

Table S5

Summary of multiple linear regression models predicting the 12-item CU scale at age 5.0 years (unadjusted for confounders)

|  |  | Model R^2^: .12 | | | |
| --- | --- | --- | --- | --- | --- |
| Variable | | ΔR^2^ | p | β | p |
| Block 1 | | .02 | .058 |  |  |
|  | Maternal positivity |  |  | -.08 | .253 |
|  | Maternal praise |  |  | -.11 | .099 |
|  | Maternal negativity |  |  | .14 | .045 |
|  | Community violence |  |  | -.01 | .868 |
| Block 2 | | .11 | <.001 |  |  |
|  | Community violence X maternal positivity |  |  | -.40 | <.001 |
|  | Community violence x maternal praise |  |  | .01 | .908 |
|  | Community violence X Maternal negativity |  |  | .16 | .140 |

Table S6

Summary of multiple linear regression model predicting the 12-item CU scale at age 5.0 from observed maternal positivity and praise and their interaction with exposure to community violence (adjusted for confounders, age 3.5 CU traits and age 5.0 ODD). Total model R^2^= .36

|  | Variable | ΔR^2^ | p | β | p |
| --- | --- | --- | --- | --- | --- |
| Block 1 |  | .35 | <.001 |  |  |
|  | Age 3.5 CU traits |  |  | .17 | .007 |
|  | Age 5.0 ODD |  |  | .42 | <.001 |
|  | Pacific region |  |  | -.06 | .346 |
|  | Caribbean region |  |  | -.08 | .207 |
|  | Mother age |  |  | .05 | .430 |
|  | Low income/low education |  |  | -.09 | .004 |
|  | Male sex |  |  | -.03 | .599 |
|  | Age 5.0 maternal depression |  |  | .19 | .004 |
| Block 2 |  | .03 | .073 |  |  |
|  | Maternal positivity |  |  | .03 | .629 |
|  | Maternal praise |  |  | -.08 | .159 |
|  | Maternal negativity |  |  | .13 | .021 |
|  | Community violence |  |  | .02 | .694 |
| Block 3 |  | .03 | .031 |  |  |
|  | Community violence X maternal positivity |  |  | -.21 | <.001 |
|  | Community violence x Maternal praise |  |  | .01 | .944 |
|  | Community violence X Maternal negativity |  |  | .08 | .421 |

Table S7

Summary of multiple linear regression model predicting the 12-item CU traits scale at age 5.0 in community violence exposed and no community violence exposed groups, adjusted for confounders, age 3.5 CU traits and age 5.0 ODD

|  |  | No community violence exposure  Total model R^2^: .24 | | | | Community violence exposed  Total model R^2^: .58 | | | |
| --- | --- | --- | --- | --- | --- | --- | --- | --- | --- |
|  | Variable | ΔR^2^ | p | β | p | ΔR^2^ | p | β | p |
| Block 1 |  | .27 | <.001 |  |  | .54 | <.001 |  |  |
|  | Age 3.5 CU traits |  |  | .20 | .018 |  |  | .18 | .033 |
|  | Age 5.0 ODD |  |  | .34 | <.001 |  |  | .36 | <.001 |
|  | Pacific region |  |  | -.11 | .194 |  |  | -.10 | .249 |
|  | Caribbean region |  |  | -.09 | .301 |  |  | -.10 | .277 |
|  | Mother age |  |  | .12 | .184 |  |  | .12 | .181 |
|  | Low income/low education |  |  | -.11 | .202 |  |  | -.11 | .184 |
|  | Male sex |  |  | .02 | .855 |  |  | .06 | .488 |
|  | Age 5.0 maternal depression |  |  | .20 | .028 |  |  | .20 | .031 |
| Block 2 |  | .04 | .071 |  |  | .10 | .001 |  |  |
|  | Maternal positivity |  |  | .18 | .032 |  |  | -.34 | .002 |
|  | Maternal praise |  |  | -.09 | .277 |  |  | -.10 | .689 |
|  | Maternal negativity |  |  | .10 | .213 |  |  | .17 | .013 |

**Simple slopes calculation for the exposed and unexposed groups**

The simple slope in the children exposed to community violence was significant and negative in direction (simple slope = -0.77 (0.35), t=-2.23, p=0.027) and significant but positive in direction in the unexposed children (simple slope = 0.48(0.24), t=1.99, p=0.048).
